# Oxidative Stress in the Pathophysiology of Kidney Disease: Implications for Noninvasive Monitoring and Identification of Biomarkers

**DOI:** 10.1155/2020/5478708

**Published:** 2020-01-23

**Authors:** Marianna Gyurászová, Radana Gurecká, Janka Bábíčková, Ľubomíra Tóthová

**Affiliations:** ^1^Institute of Molecular Biomedicine, Faculty of Medicine in Bratislava, Comenius University, Bratislava, Slovakia; ^2^Institute of Medical Physics, Biophysics, Informatics and Telemedicine, Faculty of Medicine in Bratislava, Comenius University, Bratislava, Slovakia; ^3^Department of Clinical Medicine, University of Bergen, Bergen, Norway

## Abstract

Kidney disease represents a serious global health problem. One of the main concerns is its late diagnosis, only feasible in a progressed disease state. The lack of a clinical manifestation in the early stages and the fact that the commonly measured parameters of renal function are markedly reduced only during advanced stages of the disease are the main cause. Changes at the molecular level of the kidney tissue occur even before nitrogenous substances, such as creatinine and urea, start to accumulate in the blood. Renal proximal tubules contain a large number of mitochondria and are critical for the energy-demanding process of reabsorption of water and solutes. Mitochondria are the largest producers of oxygen radicals, which, in turn, increase the susceptibility of kidneys to oxidative stress-induced damage. Free radicals and prooxidants produced during acute or chronic kidney injury may further aggravate the course of the disease and play a role in the pathogenesis of subsequent complications. Prevention might be the solution in CKD, but patients are often reluctant to undergo preventive examinations. Noninvasive markers and the possibility to obtain samples at home might help to increase compliance. This review will provide an overview of the possible uses of markers of oxidative status in noninvasive biofluids in patients with renal disease.

## 1. Introduction

Kidney disease is a worldwide health burden with a high economic cost, which includes costs for physician visits, emergency department visits, dialysis, drug cost, etc. [1–3]. It is estimated that annual medical costs for a patient with chronic kidney disease are approximately 20 thousand USD [4]. Kidney diseases are divided into two main categories, acute kidney injury (AKI) and chronic kidney disease (CKD). AKI has a fast progression and is characterized by a loss of kidney function, which leads to the accumulation of toxic end products of nitrogen metabolism and creatinine in the blood of the patient. The estimated prevalence of AKI is about 20–200 in a million people worldwide, while around 2 million people worldwide die of AKI each year [5]. CKD develops over a longer period of time and is characterized by a gradual reduction in kidney function. CKD of all stages is present in between 7 and 12% of patients in different regions of the world [6]. The global prevalence of both AKI and CKD is on the rise, partially because of the population aging, but the observed rise can be associated with the increase in hypertension and diabetes as well [6]. Moreover, CKD is a serious independent risk factor for cardiovascular disease, while AKI patients are at an increased risk of developing either de novo CKD or exacerbation of an underlying CKD, leading to end-stage renal disease (ESRD) and the need of renal replacement therapy [5, 6]. The search for screening methods allowing earlier detection and more effective disease monitoring of CKD is still ongoing. It is important to focus the research on a better understanding of the risk factors of CKD progression and more effective and less costly therapies of CKD in the later stages [7]. Renal tubule cells are rich in mitochondria, because the reabsorption of solutes is energy demanding. This makes kidney cells especially vulnerable to oxidative stress and damage [8–10]. Free radicals and prooxidants produced during the development of AKI and CKD can aggravate the injury and could play a role in the onset of severe complications in distant organs that are often observed in AKI and CKD, such as cardiovascular diseases and neurological complications [11, 12]. Several markers of oxidative stress can be measured in the serum or tissue to evaluate and monitor the disease state and progression [13]. Although serum represents a relatively stable environment to measure systemic biomarkers, blood collection can be stressful to patients. Hence, alternative biofluids are being investigated as a potential biomarker source. Both saliva and urine collection is inexpensive, easy to obtain in sufficient volumes, fast, and without the risk of vessel injury or infection [14]. The use of these alternative biofluids in clinical practice would provide practitioners with an easier means of disease monitoring.

## 2. Biomarkers of Oxidative Stress

Several comprehensive review articles regarding the physiological and pathophysiological roles of reactive oxygen species (ROS) and antioxidants in health and disease were published recently [15–18]. The aim of this review is to focus and designate current knowledge on oxidative stress biomarkers obtained noninvasively that could be used in patients with renal disease. ROS and reactive nitrogen species (RNS) are involved in a number of signaling pathways regulating cell growth and differentiation, mitogenic responses, extracellular matrix production and breakdown, apoptosis, oxygen sensing, and inflammation [19]. Besides their regulatory function, ROS and RNS are also a part of the immune system defense response against pathogenic microorganisms. On the other hand, antioxidants inhibit the formation of free radicals and prooxidants. A mechanism called antioxidant adaptation is responsible for the signal formation and transport of the appropriate antioxidant to the site of excessive free radical and prooxidant production [20]. Antioxidant enzymes, for example, glutathione peroxidase, glutathione S-transferase, and phospholipid hydroperoxide glutathione peroxidase, decompose lipid hydroperoxides to alcohols, and glutathione peroxidase and catalase also reduce hydrogen peroxide to nontoxic substances [21]. Hydrophilic or lipophilic endogenous radical-scavenging antioxidants act via suppression of redox chain initiation. Vitamin C, uric acid, bilirubin, albumin, and thiols are hydrophilic, while vitamin E and ubiquinol are lipophilic radical-scavenging antioxidants. Proteolytic enzymes and peptidases in cytosol and mitochondria can recognize and degrade proteins damaged by oxidation and prevent their accumulation, while glycosylases and nucleases repair DNA damaged by oxidation. In addition, oxidative stress is a state that occurs when free radicals and oxidants increase to the point where they overpower the radical-scavenging mechanisms of a cell [22]. However, this also means that both excess and/or an insufficient amount or activity of antioxidants may also cause oxidative stress [23–25]. The result of such stress is damage to the macromolecules such as lipids, proteins, complex carbohydrates, or nucleic acids also known as oxidative damage.

Direct analysis of free radicals in an *in vivo* system is hampered by many complications, including their very short lifespan and technical issues with their measurement. The measurement of antioxidants is problematic, since a large number of various antioxidants exist. This makes their determination time consuming and costly. Additionally, since a variety of technical equipment is needed, one laboratory would not be able to measure all antioxidants. Total antioxidant capacity (TAC) and ferric reducing ability of plasma (FRAP) measuring antioxidant power as a whole along with an indirect way of measuring oxidative stress through by-products and/or end products of oxidation reactions is more feasible. Lipid peroxidation generates a wide range of products, which can be used as biomarkers [26, 27]. Reactive aldehydes, such as malondialdehyde (MDA), can easily react with proteins to form advanced lipoxidation end products [28]. Carbonyl derivatives of amino acid residues lysine, proline, threonine, and arginine can be used as markers of protein oxidation. Advanced oxidation protein products (AOPP) are widely established markers of protein oxidation [29]. Similarly, advanced glycation end products (AGEs) are formed by reaction of carbonyl substances such as carbohydrates and proteins [30]. ROS and RNS can also damage nucleic acids, generating pyrimidine and purine base adducts. 8-oxo-2′-deoxyguanosine is thought to be the most representative product of oxidative modifications of DNA and can correlate with the level of oxidative DNA damage in the whole body [31]. Due to the single-strand nature of RNA, its repair is not possible. The most extensively studied RNA product modified by oxidation and a commonly measured marker of oxidative RNA damage is 7,8-dihydro-8-oxoguanosine [32]. It should be noted that because of ubiquitous and nonspecific nature of oxidative stress, it is always advantageous to measure a whole panel of biomarkers instead of a single parameter, as this reduces a possibility of relying on one false positive or negative result [26, 33].

## 3. Oxidative Stress in the Pathophysiology of Acute Kidney Disease

Oxidative stress is considered an important player in the pathophysiology of both, AKI and CKD. AKI is characterized by an abrupt loss of kidney function (within a week) resulting in an accumulation of toxic end products of nitrogen metabolism and creatinine in the blood, decreased urine output, or both. AKI can develop prerenally, as a consequence of decreased renal blood flow, on a renal level, caused by damage to the renal parenchyme, or postrenally, by the obstruction of urine flow from the renal collecting system or ureters [34, 35]. On a cellular level, the pathophysiology of AKI is characterized by complex interactions between immune cells causing inflammation and mediators with kidney cells [36]. The following reparatory process leads to either the restoration of kidney function or profibrotic phenotype that results in chronic kidney disease.

Hypoperfusion caused by decreased blood flow to the kidneys is the most common cause of AKI [37]. Such prerenal AKI may or may not result in a cellular injury manifested as ischemic acute tubular necrosis. The highly metabolically active proximal tubular cells and medullary thick ascending limb cells are the primary targets in such injury due to their high oxygen demands [38]. Following injury, the epithelial cells undergo structural changes or cell death, triggering endothelial activation and the infiltration of cells releasing inflammatory mediators [39]. Ischemia and reperfusion are also activators of oxidative stress, with mitochondria being the primary source of ROS in this setting [40]. During ischemic injury, ROS, such as the hydroxyl radical peroxynitrite and hyperchlorous acid, are generated. At the same time, antioxidant enzymes, such as superoxide dismutase (SOD), catalase, and glutathione reductase, are depleted. This has been shown in renal tissue after both renal ischemia and nephrotoxicity [41–43]. Ischemic injury also upregulates the expression of proinflammatory cytokines and recruits phagocytes that also generate ROS. In turn, ROS influence the vasoconstriction and renal vascular resistance [44, 45].

Sepsis-induced kidney injury is a result of both prerenal (hemodynamic changes, endothelial dysfunction) and intrarenal (inflammatory infiltration and renal parenchyma damage, intraglomerular thrombosis, and tubular obstruction) causes, but the exact cascade of events is not yet entirely known [46]. ROS are involved in the development of sepsis-induced kidney injury on multiple levels. Inflammation-induced upregulation of inducible nitric oxide synthase leads to production of excessive nitric oxide (NO), and that in turn uncouples endothelial NO synthase generating highly reactive superoxides by oxidation of oxygen [36]. In addition, the excessive NO competes with SOD and reacts with superoxide radicals creating peroxynitrite, which has a direct damaging effect on tubular cells [47, 48]. Finally, because the expression of inducible NO synthase is heterogeneous, the concentrations of NO vary, resulting in uneven perfusion [36, 48].

Inflammatory cells are notoriously known to use ROS as a part of their modus operandi. In septic AKI, dendritic cells and neutrophils are the key players in promoting renal disease [49]. While dendritic cells contribute to oxidative stress indirectly by recruiting and activating neutrophils, activated neutrophils can undergo two different ROS-dependent mechanisms to combat immediate threats. Phagocytosis (threat engulfment and “oxidative burst” activation) and NETosis (formation of neutrophil extracellular traps) require the activation of NADPH oxidase 2 (NOX2) and the production of superoxide. A recent study showed that blocking of NOX2 and inducible NO synthase in neutrophils decreases kidney injury in a mouse model of sepsis-induced AKI [49].

Oxidative stress plays an important role in the pathogenesis of rhabdomyolysis-induced myoglobinuric AKI. In rhabdomyolysis, the porphyrin ring of myoglobin is catabolized in tubules, releasing its iron content. The released iron then participates in Fenton and Haber-Weiss reactions, where catalytic amounts of iron are enough to produce ROS. Also, the heme group in myoglobin itself can promote lipid peroxidation reactions [50].

Drug-induced tubular necrosis is an acute, intrinsic renal form of AKI. The pathophysiology includes direct toxic effect of drugs on tubular cells [51]. A common form of nephrotoxic AKI is induced by cisplatin, a chemotherapy drug used to treat a number of cancers. Cisplatin invokes increased ROS production after being activated into a highly reactive form, reacts with thiol-containing molecules, such as glutathione (GSH), and depletes them [52, 53]. A decrease in cellular antioxidants can lead to the accumulation of endogenous ROS that activates signaling pathways leading to the death of renal tubular cells. Cisplatin may also induce mitochondrial dysfunction, leading to an increase in ROS production [54], and induce ROS formation in the microsomes via cytochrome P450 enzymes [55]. Studies in critically ill patients and sepsis patients with AKI showed that elevated circulating protein and lipid oxidation products correlated with the proinflammatory and prooxidative mediators and cytokines [56]. AKI and oxidative stress hold a bidirectional relationship in critically ill patients, as oxidative stress induced in AKI contributes to further injury.

## 4. Oxidative Stress in the Pathophysiology of Chronic Kidney Disease

CKD is characterized by a reduction of kidney structure and function over a period of time, to a glomerular filtration rate below 60 ml/min/1.73 m^2^ for more than 3 months, or an albumin-creatinine ratio over 30 mg of albumin for 1 g of creatinine in urine. The common causes of CKD include diabetes mellitus, hypertension, glomerulonephritis, or polycystic kidney disease. Over the course of CKD, patients progress through several stages [34, 57]. CKD generally has a slow progression, which depends on the primary disease as well as other influencing factors, such as diet, smoking, or coexisting metabolic disease. The progression of CKD to its advanced stages was shown to be associated with a significant increase in the generation of free radicals and other prooxidants. Several studies showed that the plasma markers of oxidative stress were elevated in CKD patients, indicating increased systemic oxidative stress [58–60]. Various cellular processes can serve as a source of oxidative stress in CKD patients. The combination of oxidative stress, chronic inflammation, and endothelial dysfunction is recognized as a triad perpetuating the bidirectional vicious cycle between CKD and systemic complications [61]. The development of oxidative stress in CKD is thus entwined with the progression of the disease, both as a cause and as a consequence of CKD [62]. Impaired mitochondrial function and enhanced mitochondrial ROS have been proposed as one of the causes of elevated oxidative stress in CKD. Impaired mitochondrial function might also be the cause of the lower energy metabolism displayed by many CKD patients [63]. A study comparing conservative treatment and haemodialysis in CKD patients found that the mitochondrial respiratory system was dysregulated in CKD, and this dysregulation was associated with enhanced oxidative stress [64]. In diabetic kidney disease, mitochondrial overproduction of ROS is associated with mitochondrial dysfunction, which ultimately leads to cellular damage and disease progression [63]. Animal studies on diabetic mice have shown enhanced mitochondrial ROS in the kidneys [65–67]. One study used a GFP-based redox-sensitive biosensor specifically localized in the mitochondrial matrix to prove that the enhanced oxidative stress is generated specifically in the mitochondria of diabetic mice [68]. Inflammatory processes are important players in the development of CKD. Inflammation has been linked to oxidative stress in CKD, although the precise nature of this relationship is not yet clear. There is a correlation between renal disease and markers of inflammation such as high-sensitivity C-reactive protein, interleukin- (IL-) 6, tumor necrosis factor-*α*, and fibrinogen. These molecules may induce oxidative stress via several signaling pathways [69–71]. For example, polymorphonuclear cell neutrophils generate myeloperoxidase (MPO) and activate ROS excretion. Serum MPO was found to be associated with markers of inflammation in CKD patients [72]. Increased oxidative stress may reversely worsen inflammation, setting up a vicious circle perpetuated by the activation of a nuclear transcription factor *κ*B (NF-*κ*B), which orchestrates immune cell activation and recruitment. This way, inflammatory cytokines associated with oxidative stress promote the damage of renal tissues by inducing apoptosis, necrosis, and fibrosis and may play an important role in the pathogenesis and progression of CKD [73]. Another source of oxidative stress in CKD patients may be the presence of uraemic toxins. Uraemic toxins promote inflammation, as well as oxidative stress, by priming polymorphonuclear cells, activating IL-1*β* and IL-8 and the innate immune response. Uric acid (UA) production during purine degradation through the activity of xanthine oxidoreductase and subsequent superoxide formation promotes oxidative stress. Nevertheless, increasing evidence suggests that UA itself functions as a powerful antioxidant *in vivo* [74–77].

**Figure 1:**
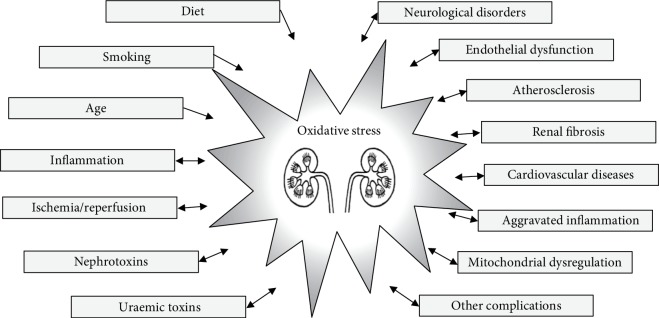
Causes and possible consequences of oxidative stress in acute and chronic kidney diseases. Increase in oxidative stress occurs as a consequence of behavioral factors and physiological and pathophysiological processes in the human body (left panel). In turn, oxidative stress contributes to the development of a variety of injuries (right panel), which may further promote oxidative stress and aggravate the initial cause, creating a “vicious circle” between oxidative stress and tissue injury.

Dysregulated metabolic waste disposal in later stages of CKD is also an important contributor to oxidative stress induction. In ESRD, renal replacement therapy with maintenance haemodialysis can aggravate oxidative stress in each session, due to ROS excretion by phagocytes on the surface of dialysis membranes. In addition, haemodialysis further exhausts the antioxidant capacity of the body [78–80]. Oxidative stress may contribute to endothelial dysfunction and can also aggravate atherosclerosis and lead to the development of cardiovascular disease or various malignancies in ESRD patients [81–83]. Increased ROS production also induces structural changes in *β*2-microglobulin, which are associated with the incidence of amyloidosis due to inflammatory processes in CKD [84]. Other features associated with oxidative stress in CKD include anaemia, hypertension, kidney fibrosis, neurologic disorders, and accelerated aging [85, 86] as summarized in Figure 1.

## 5. Monitoring Kidney Disease via Measurement of Salivary/Urinary Markers of Oxidative Stress

Markers of oxidative stress are usually measured in plasma or serum, which are relatively stable environments for the evaluation of systemic biomarkers. However, acquisition of blood might represent a significant stress to the patient. Thus, other biofluids are being tested as alternatives to plasma. These biofluids should be easier to collect, and patients should be able to produce sufficient volumes [14]. The collection of saliva is inexpensive, fast, and noninvasive. During the collection process, the risk of vessel injury and infection is eliminated. Older, less compliant patients and young children prefer the collection of saliva. Urine could also be a promising alternative biofluid for the assessment of oxidative stress, as urine collection is practically noninvasive and can be performed with no effort, equipment, or trained staff. Urine samples can also be obtained in extensive volumes, many times in one day. In addition, some argue that urine could be a better environment for the assessment of oxidative stress markers than plasma, as its metal-containing organic and inorganic compound content is lower. Due to the lower levels of ROS promoters, urine is less prone to artificial increase of oxidative stress markers during sample collection and storage [87]. Urine and saliva samples could also be collected at patients' homes, because they can be stored and transported without much effort. Although it is feasible to analyze a wide range of biomarkers in urine or saliva, the use of these biofluids in clinical practice is limited, due to a high intra- and interindividual variability of the markers (Table 1) [88]. Up to date, only a few clinical studies have been focused on salivary/urinary markers of oxidative stress in relation to kidney diseases. They are summarized in Table 2.

### 5.1. Nitric Oxide

NO is involved in many normal physiological processes, as well as in oxidative stress induction under pathological circumstances. Studies confirmed that NO is increased in saliva of CKD patients of moderate and predialysis stage when compared to controls. On the other hand, the concentration of NO is lower when compared to patients with CKD on haemodialysis [89, 90]. Even more interesting, the salivary concentrations of NO in ESRD patients on haemodialysis decreased significantly after dialysis [90, 91]. Although it seems that NO is a suitable marker with rapid salivary dynamics in CKD, it should be noted that all three studies used well-defined patients with exclusion of smokers and patients with periodontal diseases. Both smoking and periodontal diseases alter oxidative stress parameters *per se* and could possibly interfere with NO measurements. Before widespread clinical utilization, these factors should be studied or at least considered. Moreover, to our knowledge, there are no studies for NO in patients with AKI.

### 5.2. Uric Acid

UA is an important free radical scavenger in saliva and represents about 70-80% of salivary scavenging capacity [92]. Clinical studies evaluating salivary UA and CKD validated UA as a suitable biomarker for monitoring the CKD progression and/or efficacy of dialysis [93, 94]. This was confirmed not only in adults but also in children. Bibi et al. observed a 22% decrease in salivary UA concentration after dialysis [94], while Ben-Zvi et al. showed approximately 65% decrease [93]. Nevertheless, the latter study included patients with ESRD and diabetes. Maciejczyk et al. showed UA to be increased 8-fold in children with CKD when compared to the control group without CKD [95]. Additionally, this was so far the only study calculating the sensitivity and specificity, being more than 60% for both.

It is worth mentioning that either unstimulated saliva obtained by passive drooling or saliva collected using the Salivette system is appropriate and does not affect UA concentrations [92]. Uncorrected or nonnormalized values of UA correlated well with serum concentrations, at least in healthy volunteers. Blood contamination and oral health status were not associated with significant change in salivary UA concentrations [96]. Age, ethnicity, and daytime also did not affect UA levels in saliva. However, males had about 40% higher concentration of UA in saliva than females. Similarly, higher UA concentration was positively and significantly associated with body mass index. Nevertheless, if the sample is properly collected, UA in saliva has great potential to be used for routine evaluation.

### 5.3. Other Markers of Antioxidant Status

Other, more complex antioxidant status markers or high-molecular antioxidants can be measured in saliva as well. When compared to UA, their relation to kidney disease is more complicated. For example, a slight decrease in TAC and SOD following dialysis in patients without diabetes was confirmed. However, in patients with diabetes, the increase of TAC and SOD was found to be insignificant [93]. Also, significantly higher activity of peroxidase and SOD in stimulated saliva, along with the lower concentration of GSH in both stimulated and unstimulated saliva of CKD children, was observed in comparison to the control group [95]. TAC, total oxidant index, and oxidative stress index were significantly higher only when measured in stimulated saliva, while oxidative damage products (AGEs, AOPP, and MDA) were significantly higher in CKD children compared to controls regardless the type of samples [95]. This study has demonstrated that the saliva collection protocol might be of importance when measuring markers of oxidative stress in relation to kidney disease. Previously, our group also showed that using different collection tubes, daytime sampling, or periodontal status could interfere with downstream measurements of markers of oxidative stress as well as antioxidant status [97–99].

Recently published study by Maciejczyk et al. [100] showed very promising results of FRAP measurement in saliva and urine of pediatric CKD patients. Based on measured FRAP concentrations in saliva, they were able not only to discriminate control patients and patients with CKD but also to distinguish early stage CKD patients with more severe stage CKD patients. Moreover, stimulated salivary FRAP significantly correlated with both serum creatinine and urea [100].

It is possible to analyze the individual antioxidants; however, due to the high number of low- and high-molecular weight antioxidant molecules present in saliva, the assessment of total antioxidant capacity seems to be more appropriate. It appears that TAC sufficiently reflects the disturbances in antioxidant defense of CKD patients with different stages of disease [100]; however, further clinical/methodological studies are needed to confirm this.

### 5.4. AOPP, AGEs, and MDA

There are not many studies measuring salivary markers of oxidative damage to lipids (MDA), proteins (AOPP), and aldehyde (AGEs) molecules that would be in relation to kidney disease. Nevertheless, all three biomarkers seem to be increased in patients with CKD, at least in children [95]. AOPP have showed the highest diagnostic value for CKD in both stimulated and nonstimulated saliva with the best sensitivity/specificity profile [95]. However, in adults, studies evaluating these markers in saliva of CKD patients are missing.

Markers of oxidative status in urine were studied in patients undergoing liver transplantation, to assess their ability to predict early AKI [101]. Despite the same baseline levels of markers in both groups, significantly higher concentrations of MDA, 6-keto-prostaglandin F_l*α*_, H_2_O_2_, and 8-iso-prostaglandin F_2*α*_ and lower concentration of SOD were observed in the group of patients that developed AKI. Differences were present both 2 hours after graft reperfusion and 24 hours after transplantation [101]. Possible predictive and diagnostic values of urinary MDA as a marker for AKI in critically sick full-term newborns were also suggested [102]. To our best knowledge, no other clinical studies were published.

## 6. Conclusion

Oxidative stress damages renal tissue and promotes inflammation leading to further tissue injury with accumulation of impaired biomacromolecules. These can be measured also in saliva and urine which are easily collectible. Especially promising are the markers UA, FRAP, and AOPP. The current literature agrees that salivary concentrations of these biomarkers correlate well with progressed stage renal disease. Within the stage of renal disease, the salivary dynamics also shadows the plasma concentrations. Nevertheless, high variability (and low specificity) of these markers in saliva or urine has prevented their usage in routine clinical practice. Sensitivity and specificity studies with determination of cut-off values are missing, apart from those in children. Future research should yield reference values that could be further developed in standardized tests for home-based monitoring of renal disease progression in patients.

## Figures and Tables

**Table 1:** Main advantages and disadvantages of blood, urine, and saliva collection and of measuring markers of oxidative stress and antioxidant status in these body fluids.

	Blood collection	Urine collection	Saliva collection
Advantages	Stable environment Collectible in sufficient amounts Established referenced values	Noninvasive, without stress Easy to collect at home Easy to collect in sufficient amounts Inexpensive and fast collection No risk of infection Low organic/inorganic metal amount: no artificial increase of oxidative stress	Noninvasive, without stress Easy to collect at home Inexpensive and fast collection No risk of infection Low organic/inorganic metal amount: no artificial increase of oxidative stress

Disadvantages	Invasive and stressful Risk of vessel injury and infection Requires equipment Requires a trained staff Time consuming Organic/inorganic metals present: artificial increase of oxidative stress	Not established (missing reference values) High variability (hydration status, gender, age, weight, local changes, etc.)	Not established (missing reference values) High variability (flow rate, hydration status, gender, age, weight, blood contamination, local changes, etc.)

**Table 2:** Overview of clinical studies evaluating the potential of salivary and/or urinary markers of oxidative stress in patients with renal diseases.

Patient	Marker	Result	Biofluid	Reference
ESRD with DM on dialysis ESRD without DM on dialysis CKD with DM CKD without DM Healthy controls	UA, SOD TAC, Px	↓ UA in saliva after dialysis in CKD patients with or without DM	Unstimulated saliva (salivary flow rate)	2007 nonsmokers [93]

Predialysis CKD patients ESRD on dialysis	UA, TAC SOD, Px	↓ UA and ↑ peroxidase in saliva of ESRD patients on dialysis vs. predialysis CKD patients	Unstimulated saliva (salivary flow rate)	2008 nonsmokers [94]

CKD patients ESRD on dialysis Healthy controls	NO	↑ NO in saliva of CKD patients and ESRD on dialysis	Saliva	2015 [89]

ESRD on dialysis (more than 6 months) Healthy controls	NO	↑ NO in saliva of ESRD patients ↓ NO in saliva after dialysis	Filtered saliva	2018 nonsmokers [91]

Pediatric patients with CKD Healthy controls	UA, CAT, SOD, Px, GSH, TAC, TOS, OSI, AGEs, AOPP, MDA	↑ Concentrations of UA, SOD, GSH, AGEs, AOPP, and MDA in unstimulated and stimulated saliva of CKD patients vs. controls Salivary AOPP—robust marker of CKD	Unstimulated saliva Stimulated saliva (salivary flow rate)	2018 [95]

ESRD patients on dialysis Healthy controls	NO	↑ NO in saliva of ESRD patients vs. controls ↓ NO in saliva after dialysis	Unstimulated saliva	2018 nonsmokers [90]

Pediatric patients with CKD Healthy controls	FRAP, UA UA-independent FRAP	↑ FRAP in stimulated saliva and urine of CKD patients vs. controls	Unstimulated saliva Stimulated saliva Urine	2019 [100]

Liver transplantation patients Baseline 2 hours after graft reperfusion 24 hours after transplantation	MDA, H_2_O_2_ 6-keto-prostaglandin F_1*α*_, 8-keto-prostaglandin F_2*α*_	↑ Concentrations of all measured markers in patients with AKI following transplantation	Urine	2014 [101]

ESRD: end-stage renal disease; DM: diabetes mellitus; CKD: chronic kidney disease; UA: uric acid; SOD: superoxide dismutase; TAC: total antioxidant status; Px: peroxidase; NO: nitric oxide; CAT: catalase; GSH: glutathione; TOS: total oxidant status; OSI: oxidative stress index; TAC: total antioxidant capacity; AGEs: advanced glycation end products; AOPP: advanced oxidation protein products; MDA: malondialdehyde.

## Abbreviations

AKI: Acute kidney injury
CKD: Chronic kidney disease
ESRD: End-stage renal disease
ROS: Reactive oxygen species
RNS: Reactive nitrogen species
NO: Nitric oxide
NOX2: NADPH oxidase 2
SOD: Superoxide dismutase
GSH: Glutathione (reduced)
UA: Uric acid
MPO: Myeloperoxidase
NF-*κ*B: Nuclear transcription factor *κ*B
MDA: Malondialdehyde
AGEs: Advanced glycation end products
AOPP: Advanced oxidation protein products
FRAP: Ferric reducing antioxidant power
TAC: Total antioxidant capacity.

## Supplementary Materials

Supplementary Materials Graphical abstract.
