# Supplementary figures and images for: Oxidative Stress in the Pathophysiology of Kidney Disease: Implications for Noninvasive Monitoring and Identification of Biomarkers
Source: Oxid Med Cell Longev. 2020 Jan 23;2020:5478708. doi: 10.1155/2020/5478708 (PMC7007944; doi:10.1155/2020/5478708)

## Graphical Abstract

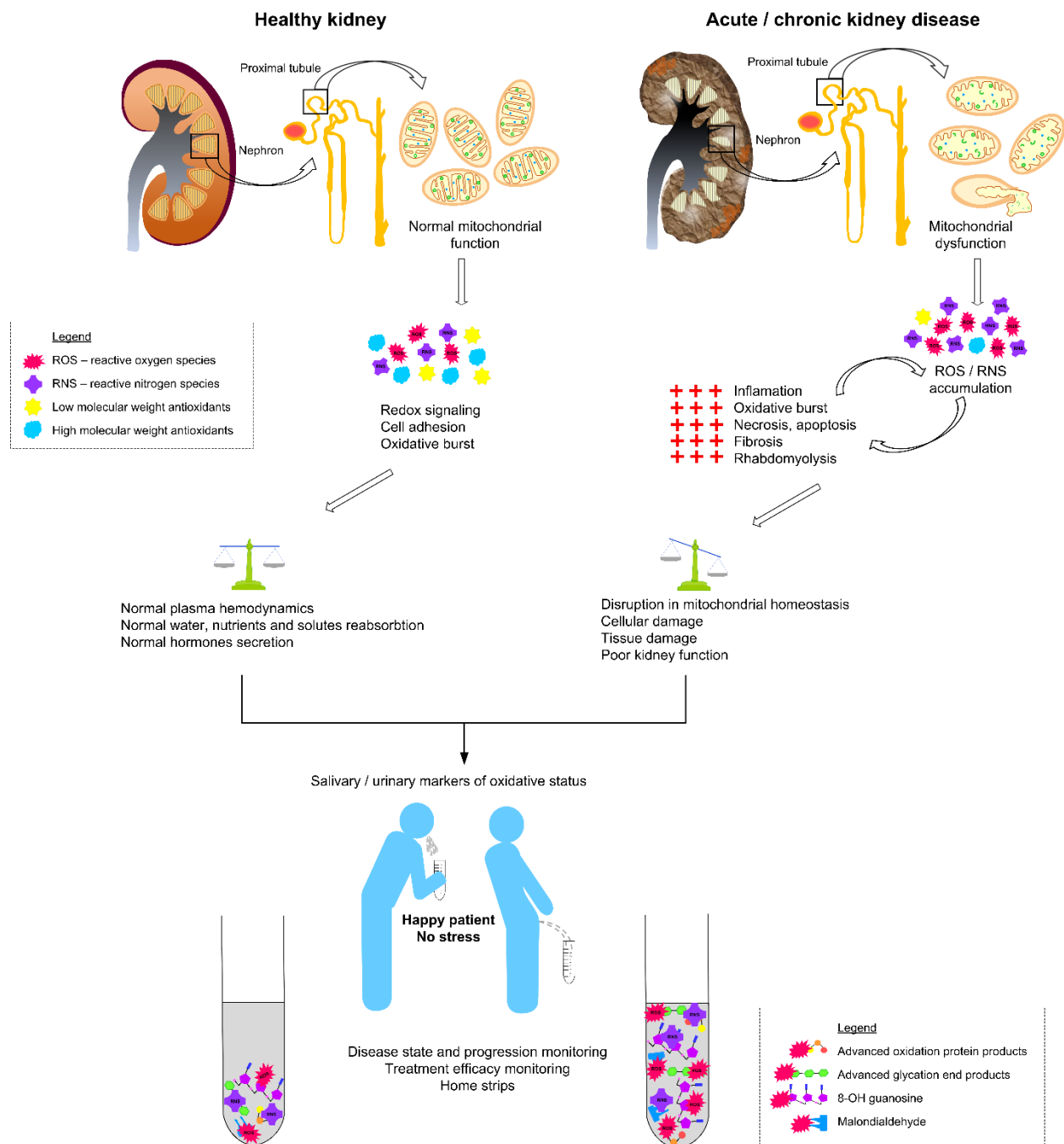

Supplement: Supplementary Materials — Graphical abstract. [file 5478708.f1.pdf]
